# A comparative study between Ketorolac and Ketoprofen in postoperative pain after uvulop alatopharyngoplasty

**DOI:** 10.1016/S1808-8694(15)30077-X

**Published:** 2015-10-19

**Authors:** Lucas Gomes Patrocínio, Marcelo de Oliveira Rangel, Gustavo Sousa Marques Miziara, Alexandre Menezes Rodrigues, José Antonio Patrocínio, Tomas Gomes Patrocinio

**Affiliations:** a Physician, Resident in the Otorhinolaryngology Unit of the Uberlandia Federal University Medical School; b Physician, Resident in the Otorhinolaryngology Unit of the Uberlandia Federal University Medical School; c Physician, Resident in the Otorhinolaryngology Unit of the Uberlandia Federal University Medical School; d Doctor on Anesthesiology, Anesthetist of the Anesthesiology Unit of the Santa Genoveva Hospital; e Full Professor, Head of the Otorhinolaryngology Unit of the Uberlandia Federal University Medical School; f Physician, Resident in the Otorhinolaryngology Unit of the Uberlandia Federal University Medical School. Otorhinolaryngology Unit of the Santa Genoveva Hospital, Uberlandia, Minas Gerais, Brazil

**Keywords:** sleep apnea, postoperative, pain, obtructive, snoring

## Abstract

Postoperative pain is a serious problem, requiring an appropriate response from the medical doctor. In otolaryngology special attention is needed after uvulopala topharyngoplasty (UP3). **Aim:** To compare the efficacy of postoperative analgesia using ketorolac and ketoprofen after UP3. **Patients and Methods:** A prospective, randomized, double-blind study was made of 24 patients that were divided into 2 groups (14 received ketorolac and 10 received ketoprofen). Pain intensity was based on an analog visual scale and the need for opioids (tramadol). **Results:** Of the 13 patients that received ketorolac, 3 (21%) required opioids; 7 of 10 (70%) patients in ketoprofen group used opioids. 12 hours after surgery, 71% of the patients that received ketorolac had mild or absence of pain. 70% of the ketoprofen users reported moderate to significant pain. 24 hours after surgery, 60% of the patients using ketoprofen reported moderate to significant pain, while 86% of the ketorolac users reported mild or absence of pain. **Conclusion:** We concluded that ketorolac is more effective compared to ketoprofen in the treatment of immediate postoperative pain after UP3, as patients using ketorolac had less pain and used opioids to a lesser degree.

## INTRODUCTION

Pain is defined as an unpleasant sensory and emotional experience resulting from, or described in terms of, true or potential tissue injury. Pain sets in motion mechanisms in the body that result in incapacity and adverse biopsychosocial effects.^1^

The intensity and expression of pain vary considerably between subjects and between animal species. It is also affected by environmental, racial, religious, cultural, and philosophical factors, and by past experience and the mental state.^1^, ^2^

Measurement of pain is essential for its treatment. Being a subjective experience, pain cannot be measured objectively by physical instruments such as those that measure weight, temperature, and blood pressure. On the other hand a variety of tools for assessing subjectivity are available that allow patients to measure the intensity of pain with little mental or physical effort. Various scales are available: the Verbal Rating Scale, the Pain Relief Ordinal Scale, the Numeric Rating Scale, the Visual Analog Scale, and the Faces Pain Scale. Any pain assessment process should be systematic and continuous, and should be reported so that there is an effective contribution to the treatment of pain.^3^

Immediate postoperative pain (during the first 24 hours) is a difficult daily problem that requires adequate support from physicians. Special attention is required for the treatment of pain in otorhinolaryngology following tonsillectomy and uvulopalatopharyngoplasty (UPPP). UPPP is a procedure done by otorhinolaryngologists as an option to treat the obstructive sleep apnea and hypopnea syndrome (OSAHS). Regardless of the technique, pain after these procedures is classified as moderate to severe.

Various analgesic drugs have been used against postoperative pain following UPPP. Ketorolac tromethamine (Toradol **®**) is a non-steroidal anti-inflammatory drug (NSAID) that has an analgesic efficacy similar to commonly used opioids, and that recently has found wide acceptance in the treatment of postoperative pain in a variety of surgical procedures. Ketorolac is used for moderate pain relief; it may be used to treat severe pain when associated with opioids, reducing the opioid dose. The advantage of this association is the reduction of opioid side effects such as respiratory depression, pruritus, urinary retention, sedation and nausea.^4^-^6^

The aim of this paper was to measure the analgesic effect of ketorolac during the immediate postoperative period following UFPF. This drug was compared with ketoprofen, another well-known anti-inflammatory drug that is also used parenterally, to assess the analgesic effect and the need for opioids.

## PATIENTS AND METHOD

This trial was a prospective, randomized, blinded study that included 24 patients aged between 21 and 60 years that underwent UPPP between June 2004 and June 2005.

A single intervention associated septoplasty, partial inferior turbinectomy, and tonsillectomy in all of the cases. Patients with a history of allergic reactions to NSAIDs, asthma, gastroduodenal ulcers, or bleeding disorders were excluded. Also excluded were patients in which other procedures such as glossectomy, blepharoplasty, ritidoplasty, or sinusectomy were added.

The intensity of postoperative pain was assessed in these patients 12 and 24 hours after the surgical procedure. Assessment of pain intensity was based on the faces pain scale and a 6-point verbal rating scale.

The trial was a comparative study of the analgesic effect of ketorolac tromethamine (30 mg EV every 8 hours, beginning at induction of anesthesia) and ketoprofen (100 mg EV, every 12 hours), and the need for rescue medication with an opioid (tramadol).

The analgesic drug for each patients was chosen randomly based on the last digit of their medical chart number (if odd - Ketorolac; if even - Ketoprofen).

Before surgery, the medical team handed a form over to the patient and family member containing their name, age, chart number, type of surgical procedure, analgesic medication used, and the visual pain scale; instructions on how to fill in the form correctly was given at this time. Furthermore, patients were told that if excessive pain was felt, they should call the nurse; the nurse would inform the medical team and administer tramadol.

UPPP was done under general anesthesia to which was added local infiltration of bupivacaine 0.5% with adrenalin 1:80.000 on the base of the uvula and the palatine arch. Oblique bilateral incisions were made on the superior portion of the tonsillar pillars, followed by partial sectioning with electrocautery of the uvula. Vicryl 3.0 sutures were made on the posterior and anterior pillars.

Results were assessed statistically using the chi-squared and Student’s t-test. The trial was approved by the Research Ethics Committee of the institution and given the number 025/04.

## RESULTS

UPPP was done on 24 patients, of which 14 received ketorolac and 10 received ketoprofen. The groups were compared according to gender and number of patients. There was no significant difference between the number of patients in each group, but males predominated in both groups (Table 1).

**Table 1:** Distribution of patients undergoing uvulopalatopharyngoplasty according to gender and medication.

Drug used	Male	Female	Total
Ketoprofen	9 (90%)	1 (10%)	10
Ketorolac	11 (79%)	3 (21%)	14

Total	20 (83%)	4 (17%)	24

p < 0,05

Twenty one percent of 14 patients that received ketorolac used opioids postoperatively. Seventy percent of 10 patients that received ketoprofen used opioids for the treatment of postoperative pain following UPPP (p < 0.01) (Table 2).

**Table 2:** Distribution of patients undergoing uvulopalatopharyngoplasty according to the need for postoperative rescue medication (tramadol).

Drug used	Use of tramadol	Non-use of tramadol	Total
Ketoprofen	7 (70%)	3 (30%)	10
Ketorolac	4 (21%)	10 (79%)	14

Total	11 (46%)	13 (54%)	24

p < 0,05

**Chart 1:**
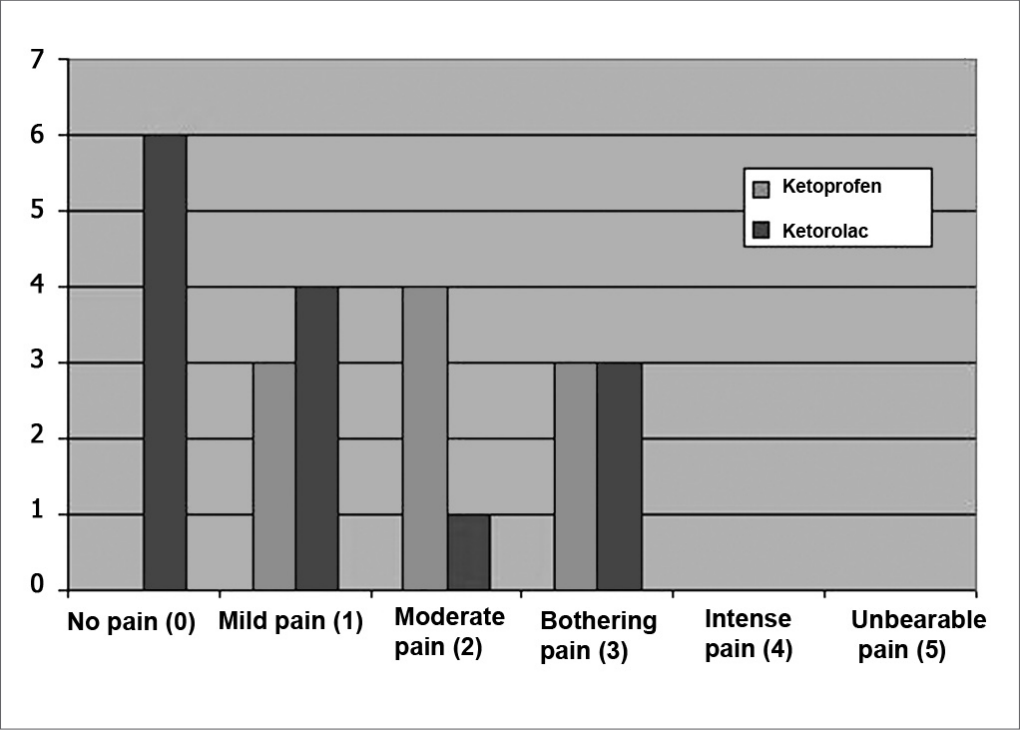
Distribution of patients undergoing uvulopalatopharyngoplasty according to the intensity of pain 12 hours postoperatively.

**Chart 2:**
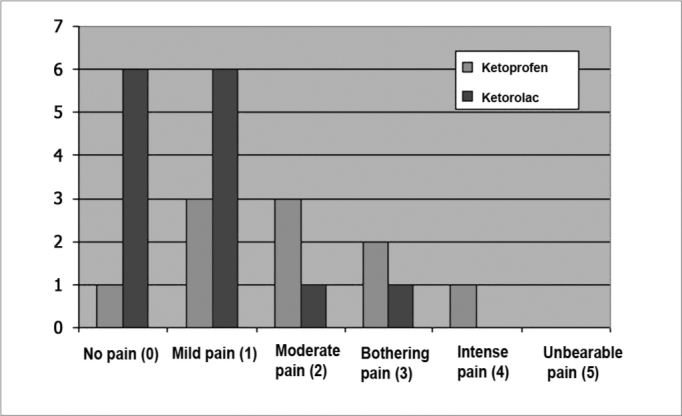
Distribution of patients undergoing uvulopalatopharyngoplasty according to the intensity of pain 24 hours postoperatively.

The visual analog scale and the verbal rating scale revealed significant differences in the analysis of pain intensity 12 and 24 hours after surgery. Twelve hours postoperatively, mild or absent pain was present in 71% of patients that were medicated with ketorolac, and moderate or annoying pain was present in 70% of patients that were medicated with ketoprofen (Table 3 and Chart 1) (p < 0.05). Twenty-four hours postoperatively, moderate or annoying pain was reported by 60% of patients that were medicated with ketoprofen, and 86% of patients that were medicated with ketorolac reported mild or absent pain (Table 4 and Chart 2) (p < 0.05).

**Table 3:** Distribution of patients undergoing uvulopalatopharyngoplasty according to the intensity of pain 12 hours postoperatively.

Drug	Absence of pain	Mild pain	Moderate pain	Annoying pain	Severe pain	Unbearable pain	Total
Ketoprofen	0	3 (30%)	4 (40%)	3 (30%)	0	0	10
Ketorolac	6 (43%)	4 (29%)	1 (7%)	3 (21%)	0	0	14

Total	6	7	5	6	0	0	24

p < 0,05

**Table 4:** Distribution of patients undergoing uvulopalatopharyngoplasty according to the intensity of pain 24 hours postoperatively.

Drug	Absence of pain	Mild pain	Moderate pain	Annoying pain	Severe pain	Unbearable pain	Total
Ketoprofen	1 (10%)	3 (30%)	3 (30%)	2 (20%)	1 (10%)	0	10
Ketorolac	6 (43%)	6 (43%)	1 (7%)	1 (7%)	0	0	14

Total	7	9	4	3	1	0	24

p < 0,05

There were no perioperative or postoperative complications.

## DISCUSSION

Current treatment of pain is based on the concept of balanced or multimodal analgesia in which pharmacologically different drugs are used to obtain synergy and a lower rate of undesired effects.^7^ Careful management of postoperative analgesia results in a reduced hospital stay, earlier recovery of function (especially food intake), and increased success of the surgery in general.8 In our trial the use of opioids was significantly lower in the group that received ketorolac compared to ketoprofen (p < 0.05). Furthermore, reported subjective pain was significantly lower at 12 and 24 hours after surgery (p < 0.05).

The efficacy of ketorolac has already been demonstrated in other trials investigating pain relief after tonsillectomy. Rusy et al. (1995) found that ketorolac was more effective than acetaminophen in the treatment of postoperative pain following tonsillectomy in children.^9^ Forrest et al. (1997) reported that ketorolac was more advantageous compared to opioid analgesics in the control of post-tonsillectomy pain. This was due to lower rates of sedation, nausea, vomiting, and respiratory depression and a similar level of analgesia when using ketorolac, compared to commonly used opioids.^10^

Tarkkila and Saarnivaara (1999)^11^ compared ketorolac, ketoprofen, and diclofenac postoperatively following elective tonsillectomy, and reported a lower use of opioids, improved pain control, and a similar complication rate compared to placebo. These findings are similar to those we found in our trial. Until now no papers had been published evaluating ketorolac after UPPP.

O’Donovan et al.^12^ (1994) and Pernice et al.^13^ (2001) demonstrated that ketorolac is also effective in controlling postoperative pain and reducing urinary retention following hemorrhoidectomy. According to Carney et al. (2001)^5^ ketorolac reduced the use of opioids and lowered the morbidity during the first 48 hours after pediatric surgery. Furthermore, patients receiving ketorolac did not present increased bleeding or kidney toxicity compared to the group that received morphine only. Shende and Das (1999)^14^ reported a lower rate of vomiting and pain when using ketorolac postoperatively after strabismus surgery in children compared to the placebo group. The rate of postoperative bleeding was similar to that found in traditional treatments.

Many papers have shown that ketorolac used in subjects below 65 years, at an average dose of 100 mg/day or less, during 5 days or less, was not associated with a detectable increased risk of gastrointestinal or surgical wound bleeding. ^4^ We also found that postoperative bleeding was not increased in our sample.

## CONCLUSION

We concluded that ketorolac is more effective compared to ketoprofen to treat immediate postoperative pain following UPPP. Patients medicated with ketorolac had pain of lower intensity compared to those treated with ketoprofen, and required less opioids as supplementary treatment.
